# Effectiveness of Team and Organisational Level Workplace Interventions Aimed at Improving Sustainable Employability of Aged Care Staff: A Systematic Review

**DOI:** 10.1007/s10926-022-10064-5

**Published:** 2022-09-23

**Authors:** Ceciel H. Heijkants, Astrid de Wind, Madelon L. M. van Hooff, Sabine A. E. Geurts, Cécile R. L. Boot

**Affiliations:** 1 Behavioural Science Institute, Radboud University, Nijmegen, The Netherlands; 2 Department of Public and Occupational Health, Coronel Institute of Occupational Health, Amsterdam Public Health Research Institute, Amsterdam UMC, University of Amsterdam, Amsterdam, The Netherlands; 3 Department of Public and Occupational Health, Amsterdam Public Health Research Institute, Amsterdam UMC, VU University, Amsterdam, The Netherlands; 4 Present Address: Thomas van Aquinostraat 4, room 04.362, 6525GD Nijmegen, The Netherlands

**Keywords:** Program evaluation, Workplace, Geriatric nursing, Occupational health

## Abstract

*Purpose* The sustainable employability of healthcare professionals in aged care is under pressure, but research into the effectiveness of interventions aimed at improving employees’ sustainable employability is scarce. This review therefore aimed to investigate the effectiveness of workplace interventions on sustainable employability of healthcare professionals in aged care. *Methods* A systematic literature search was performed. Studies were included when reporting about the effect of an intervention at work in an aged care setting on outcomes related to one of the three components of sustainable employability (i.e. workability, vitality, employability). The methodological quality of each study was assessed and a rating system was used to determine the level of evidence. Additionally, a sensitivity analysis was performed, accounting for the match between the intervention’s focus and the targeted component of sustainable employability. *Results* Current review includes 32 interventions published between 1996 and 2019. Interventions covered learning and improving skills, changing the workplace, and exercising or resting. The initial analysis showed a strong level of evidence for employability and insufficient evidence for workability and vitality. The sensitivity analysis revealed strong evidence for the effectiveness of interventions addressing either employability or workability, and insufficient evidence for vitality. *Conclusions* Evidence for workplace interventions on sustainable employability of healthcare professionals in aged care differed. We found strong evidence for effects of workplace interventions on employability and for those directly targeting workability. Evidence for effects of interventions on vitality was insufficient. The alignment of the interventions to the targeted component of sustainable employability is important for effectiveness.

**Supplementary Information:**

The online version contains supplementary material available at 10.1007/s10926-022-10064-5.

## Background

Retaining healthcare professionals in aged care for their profession is an important yet challenging task nowadays. While the aging population increases the demand for care for older adults, the number of caregivers relative to older people has stagnated in most countries since 2011 [1]. This stagnation originates from the difficulty to attract young people and the challenge to retain current staff [1]. Job characteristics, like low compensation, high physical and emotional demands, heavy workload, scheduling challenges, insufficient supervision and limited training and career advancement prospects are related to job dissatisfaction and high turnover [2–6], which reinforces the difficulties for those remaining in the job [1, 7–10].

With many staff leaving the profession, sustainable employability of the workforce is a growing concern [11]. Sustainable employability has been defined by van der Klink et al. [12] as opportunities and conditions needed for employees to ‘make a valuable contribution through their work, now and in the future, while safeguarding their health and welfare’ (p. 74). In scholarly literature, three components have been distinguished to reflect an individual’s sustainable employability, i.e. workability, vitality and employability [13–15]. *Workability* has been described as the physical, mental and social capacity needed to deal with work demands [16]. Workability is therefore closely related to health, which is not limited to the absence of disease but refers to a complete state of physical, mental and social wellbeing [17]. *Vitality* has been defined as experiencing levels of energy and motivation [15], as being intrinsically motivated [18], and also as a state of both high psychological well-being and physical health [19]. *Employability* refers to the ability to adequately perform various tasks and to function optimally at work, now and in the future [15]. Taken together, workability mainly focuses on the health and functional capacity of employees, vitality mostly concerns energy and motivation, and employability focuses on employees’ knowledge and competences [20].

Given the growing general interest in sustainable employability, it is not surprising that previous reviews have attempted to shed light on the effectiveness of interventions aimed at improving sustainable employability of employees. These reviews show that there (a) is insufficient evidence for the effectiveness of interventions on measures of sustainable employability specifically among aging employees [21], (b) is moderate-quality evidence for the effectiveness of workplace interventions on workability [22], and (c) are significant positive effects in a minority of interventions when aimed at the capabilities of an employee, i.e. employability [23]. Methodological limitations like small sample sizes and/or lack of high quality interventions hamper definite conclusions about the effectiveness of these interventions [21, 23].

From these previous reviews it is still unclear what effects interventions could have on the broad spectrum of sustainable employability (i.e., workability, vitality and employability), and for healthcare professionals caring for older adults in particular. Even more so because included interventions are mostly directed at the individual level. Interventions implemented on team- or organisational level might be more promising, since comprehensive interventions on all levels of the organisation are amongst the most effective to change wellbeing inhibiting factors at work [24]. Workplace interventions on the team- or organisational level seem to be especially promising in health care, where trust in management and teamwork appear to contribute to sustainable employability over time [11]. There is also the additional benefit of reaching larger groups of employees with team- or organisational level interventions [25].

Organisations providing care for older adults are urgently looking for ways to improve the sustainable employability of their staff. However, they lack an integrative overview of effective interventions to make evidence-based decisions. The present systematic review aims to contribute to a better understanding of the effectiveness of interventions aimed at improving sustainable employability by a) incorporating a broad conceptualisation of sustainable employability that includes all three components (workability, vitality and employability), b) including interventions at the team- and organisational level (referred to as workplace interventions), and c) tailoring the search to a sector which is specifically in need of a sustainable workforce, namely aged care. Our review will not only shed more light on the effectiveness of workplace level interventions aimed at improving sustainable employability, but will also guide organisations providing care for older adults in their search towards an approach to attain a sustainable workforce that is so highly needed, now and in the future.

## Methods

We conducted a systematic review guided by the principles of the Preferred Reporting Items for Systematic Reviews and Meta-Analyses (PRISMA) [26]. We registered the review protocol in the International Prospective Register of Systematic Reviews (PROSPERO ID:161,999).

### Search Strategy

Together with an information specialist we designed our search strategy that involved searches in five databases: Embase, CINAHL, Medline, PsycINFO, and Web of Science. Our search strategy consisted of keywords related to the population of interest (e.g. caregivers, care providers or nurses), the setting (e.g. long-term care, elderly care), the context (e.g. workplace or job), the intervention (e.g. intervention, training or program) and design (e.g. controlled study or randomised controlled trial). We did not specify keywords related to outcomes, because we aimed to include a broad range of outcome measures related to our operationalisation of sustainable employability. The search was restricted to English language journal articles and covered all articles available at the time of the literature search (January 20th 2020). The full search strategy can be found in the supplementary materials.

### Operationalisation of Sustainable Employability

In this study we define an individual’s sustainable employability according to three components, i.e. workability, vitality and employability [14, 15, 27]. Conceptually there is some overlap between the three components, especially between vitality and workability. Both workability and vitality have been described in terms of psychological well-being and physical health [16, 19]. In order to better distinguish between the three components of sustainable employability in this review, we define workability in terms of physical health and functional capacity of employees, vitality in terms of mental health, energy and motivation, and employability in terms of employees’ knowledge and competences [20]. We made a distinction between physical health (workability) and mental health (vitality), since the latter has a closer relationship with mental processes like feeling energetic and intrinsically motivated [18, 28]. Table 1 shows our operationalisation of the three components and corresponding example outcome measures.

**Table 1:** Operationalisation of sustainable employability components and related example outcome measures

Sustainable employability component	Operationalisation	Example outcome measures
Workability	The extent to which employees’ physical health and functional capacities (un)able them to meet the requirements of their work	Muscle strength or injury
Vitality	The extent to which employees’ mental health, energy and motivation (un)able them to work long and tirelessly	Well-being, burnout or engagement
Employability	The extent to which employees’ knowledge and competences (un)able them to adequately continue working	Knowledge, skills or confidence

### Inclusion and Exclusion Criteria

Studies were eligible for inclusion in this review if they evaluated an intervention at work that targeted one or more outcomes related to one or more components of healthcare professionals’ sustainable employability. Because we were interested in workplace interventions that were implemented on the team- or organisational level, interventions focusing on individuals, as well as national or governmental policy were excluded. The intervention should have been implemented in a setting in which older people (often called ‘residents’) live or are taken care of (e.g. nursing homes, dementia special care units or home care). The study had to report on outcomes measures related to workability, vitality and/or employability of the care staff. In terms of design we included studies that were randomised or non-randomised controlled trials reporting at least two time points (pre- and post-intervention). The analytical strategy had to take into account the effect of group over time (e.g. interaction effect or adjusting for baseline value of outcome measure). Only primary quantitative studies were eligible for inclusion; qualitative studies, meta-analyses or theoretical papers were excluded.

### Study Selection

**Fig. 1:**
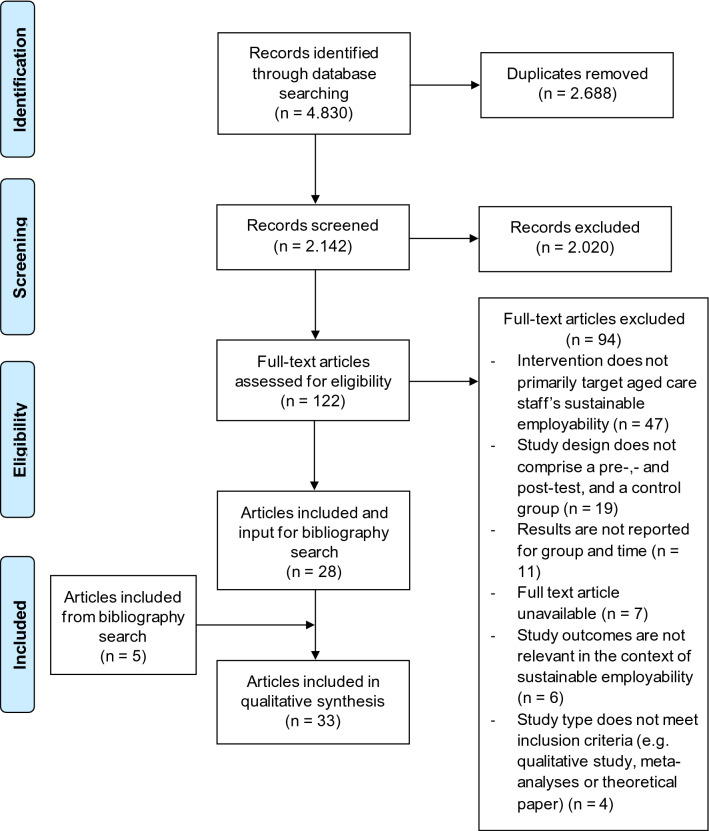
PRISMA flow diagram

An overview of the study selection based on the PRISMA flow diagram [29] is depicted in Fig. 1. After a first draw from the databases, all duplicates were removed. Next, the first and second author used Rayyan [30], an online tool for systematic reviews, to assess titles and abstracts of the remaining studies based on the inclusion and exclusion criteria. After screening 20 percent of the studies, there was no more than 5 percent disagreement between the first and second author about selection of the full-text paper. The first author continued to screen the remaining titles and abstracts and discussed any doubts with the second author. For the titles and abstracts that were selected for full-text appraisal, full-text copies were retrieved, which were assessed by the two authors independently. In case of a disagreement, the third and fifth author were consulted for a final decision. The first author screened the bibliography and identified 10 additional studies of interest. Based on the inclusion and exclusion criteria the first and second author agreed to include five more studies from the bibliography search, resulting in a total of 33 studies included in the synthesis.

### Data Extraction

A custom made data-extraction form was used to extract the following data per study: author, publication year and country, study population, study design, intervention, outcome measures, significance and direction of relationships. Study outcomes were categorized into workability, vitality and employability. Results on outcome measures that did not fit into one of the three components of sustainable employability were not extracted (e.g. resident outcomes, training evaluations). The first and second author extracted the data of five articles together, after which the first author continued the extraction. The second author, and in some instances other authors, was involved in cases of uncertainty about the extracted data.

### Quality Assessment

The methodological quality of the included studies was independently assessed by the first two authors by means of the Quality Assessment Tool for Quantitative Studies developed by the Effective Public Health Practice Project [31]. This tool is suitable for the assessment of both randomised and non-randomised studies and has been used previously in other (review) studies [23, 32, 33]. The tool consists of six sections: selection bias, study design, confounders, blinding, data collection methods, and withdrawals and dropouts. Each section was assessed as ‘strong’, ‘moderate’ or ‘weak’, in consonance with the tool’s dictionary. The overall quality of the studies was either strong (i.e. no weak sections), moderate (i.e. one weak section) or weak (i.e. two or more weak sections). Any discrepancies within ratings of the two reviewers were discussed in order to reach consensus.

### Level of Evidence Rating

To our knowledge there is no existing level of evidence scheme that takes into account heterogeneity of outcome measures within single studies and heterogeneity of outcome measures within overarching components. Before we could apply an already existing rating system by Hoogendoorn et al. [34] and Van Drongelen et al. [35], we had to conduct two additional steps as this rating system does not take into account the possibility that one study contains multiple outcomes. Because we postulated these steps post-hoc, they were not included in the preregistration.

First, we looked at the statistical significance and direction of the relationships found within a study. If 50 percent or more of the outcome measures within a study were statistically significantly impacted in the same direction (p < 0.05), this was noted as ‘statistically significant impact’ in the data extraction table. This could be either a positive or negative statistical significance in relation to the component of sustainable employability at hand. If less than 50 percent of the outcomes were statistically significantly impacted, this was noted as ‘no statistically significant impact’ for that particular study on the component of sustainable employability. As most of the articles had 1, 2 or 3 outcome measures per component of sustainable employability, the 50 percent cut-off score was chosen because it yields the right balance without being too strict or too lenient in deciding upon the statistical impact of an intervention.

Subsequently, we looked at the consistency of the statistical significance and the direction of the relationships found across studies on the level of each component of sustainable employability. Here we used the cut-off score by Hoogendoorn’s et al. [34] and Van Drongelen’s et al. [35] rating scheme stating there is ‘insufficient evidence’ for an effect if less than 75 percent of the studies within a component of sustainable employability has been evaluated as having a statistically significant impact in the same direction. If this percentage is (more than) 75 percent, the findings are considered ‘consistent’.

In the last step we rated every component of sustainable employability by the scheme of by Hoogendoorn et al. [34] and Van Drongelen et al. [35] including the quality assessment rating for a final decision on evidence. The level of evidence was therefore either ‘strong’ (consistent findings in multiple high-quality studies), ‘moderate’ (consistent findings in one high-quality and/or multiple moderate-quality studies) or ‘insufficient’ (only one study available or inconsistent findings in multiple studies).

In addition, a sensitivity analysis was performed, in which we repeated the steps described above, but only including interventions directly addressing the component of sustainable employability. We have postulated this sensitivity analysis post-hoc, because we noted during data extraction that alignment between the intervention and outcome measures was not self-evident. As such, this analysis was not included in the preregistration.

## Results

A total of 33 controlled trials published between 1996 and 2019 were included in the synthesis. Two articles reported about the same intervention [36, 37], which resulted in 32 unique interventions. Studies took place in several parts of the world (i.e. Europe, Australia, USA, Asia). Seventeen interventions focused on care-related education (e.g. person-centred care, oral health care or care with minimal restraints). Ten interventions mainly focused on skills (e.g. communication skills, feeding skills, dealing with challenging behaviours). Two programs aimed at changing the workplace (e.g. work improvement or redesign, implementing ceiling lifts) and three interventions involved physical activity or resting periods for the employees (e.g. exercising or a relaxing foot massage during work). Six studies consisted of one session, all the other studies included several sessions over the course of multiple weeks or months. Table 2 shows characteristics and results of the included studies, categorized by the outcome measure of interest matching the component of sustainable employability.

**Table 2:** Descriptives and results classified by outcome measures’ relatedness to components of sustainable employability (i.e. workability, vitality or employability)

Study outcomes related to workability									
First Author	Public-ation year	Country	Study population	Study design	Intervention	Frequency/duration	Outcomes	Impact	Overall Quality Assessment
Kuske, B.	2009^a^^,b^	Germany	Nursing home staff (N = 134)	Cluster randomised controlled trial T0 = prior to intervention T1 = after the intervention T2 = 6 months follow-up	Training in dealing and interacting with people with dementia, including lectures, videotapes, handouts, brainstorming and short games	13 weekly sessions of ~ 60 min. over the course of 13 weeks	− Global physical impairment	No statistically significant impact	Strong
O'Brien, W.H.	2019^a^	USA	Nurses and nursing aides (N = 71)	Cluster randomised controlled trial T0 = prior to intervention T1 = 1 month follow-up	Acceptance and Commitment Therapy (ACT)	2 sessions of ~ 150 min. spaced 1 week apart	− Injury frequency and musculoskeletal symptoms in prior month	No statistically significant impact	Strong
Rasmussen, C.D.N.R.	2015	Denmark	Nurses' aides, kitchen and cleaning personnel, janitors (service workers) (N = 594)	Stepped wedge cluster randomised controlled trial	Multifaceted workplace intervention for low back pain	5 sessions participatory ergonomics of ~ 120 min., 2 sessions cognitive behaviour training of ~ 180 min., 12 sessions physical training of ~ 60 min. and 3 sessions for supervisors of ~ 60 min. all over the course of 3 months	↓ Lower back pain days ↓ Lower back pain intensity ↓ Lower back pain bothersomeness	Statistically significant positive impact	Strong
Dulon, M.	2009	Germany	Nurses (N = 1159)	Randomised controlled trial T0 = prior to intervention T1 = 12 months follow-up	An educational skin care programme, including advisory service	4 h seminar for senior nurses and 2 h session skin protection program (repeated until 75% of the nurses had participated)	↓ Skin symptoms	Statistically significant positive impact	Moderate
Moyle, W.	2013^a^	Australia	Nursing staff (N = 19)	Randomised controlled trial T0 = prior to intervention T1 = after the intervention	Foot massage intervention at work	Max 3 sessions a week of ~ 10 min. over the course of 4 weeks	↓ Diastolic blood pressure − Systolic blood pressure	Statistically significant positive impact	Moderate
Skargren, E.	1996	Sweden	Nurses and nursing aides (N = 90)	Controlled clinical trial T0 = prior to intervention T1 = after the intervention	Exercise program	~ 2 sessions a week of ~ 45 min. over the course of 8 weeks	↑ Cardio vascular capacity ↑ Muscle strength ↓ Musculoskeletal symptoms − Psychosomatic symptoms	Statistically significant positive impact	Moderate
Engst, C.	2005^a^	Canada	Nursing staff (N = 50)	Controlled clinical trial T0 = prior to intervention T1 = 12 month follow-up	The instalment and use of overhead ceiling lifts including a 'no-unsafe manual lift' policy	1 session of ~ 60 min	↓ Perceived risk of injury to the neck, shoulders, upper/lower back and arms/hands ↓ Discomfort	Statistically significant positive impact	Weak

Study outcomes related to vitality									
Barbosa, A.	2015 & 2016	Portugal	Nursing staff (N = 58)	Randomised controlled trial T0 = 3 weeks prior T1 = 2 weeks follow-up T2 = 6 months follow-up	A person-centred care based psychoeducational intervention, including education and support	8 weekly sessions of ~ 90 min	↓ Emotional exhaustion − Personal accomplishment − Depersonalisation − Perceived stress − Job satisfaction	No statistically significant impact	Strong
Broughton, M.	2011^b^	Australia	Nursing assistants, registered/endorsed/enrolled nurses and recreational/activities officers (N = 68)	Controlled clinical trial T0 = prior to intervention T1 = after the intervention T2 = 3 months follow-up	A DVD-based caregiver education program to support memory and communication in dementia, including a booklet summarizing information	1 session of ~ 90 min	− Satisfaction and positive rewards experienced in the caregiving role	No statistically significant impact	Strong
Fukuda, K..	2018	Japan	Staff (N = 400)	Cluster randomised controlled trial T0 = prior to intervention T1 = 1 month follow-up	An educational program on (the implementation of guidelines) on behavioural and psychological symptoms of dementia (BPSD)	1 session of ~ 120 min	↓ Severity of BPSD and the burden of it on the caregiver − Personal accomplishment − Depersonalisation − Emotional exhaustion	No statistically significant impact	Strong
Kloos, N.	2019	The Netherlands	Nursing staff (N = 165)	Cluster randomised controlled trial T0 = prior to intervention T1 = 12 weeks follow-up T2 = after the intervention	An online gamified multicomponent positive psychology intervention	8 weekly sessions over the course of 8–12 weeks	↑ Job satisfaction − Wellbeing − Work engagement	No statistically significant impact	Strong
Kuske, B.	2009 ^b,c^	Germany	Nursing home staff (N = 134)	Cluster randomised controlled trial T0 = prior to intervention T1 = after the intervention T2 = 6 months follow-up	Training in dealing and interacting with people with dementia, including lectures, videotapes, handouts, brainstorming and short games	13 weekly sessions of ~ 60 min. over the course of 13 weeks	− Personal accomplishment − Depersonalisation − Emotional exhaustion	No statistically significant impact	Strong
Mackenzie, C.S.	2003 ^b^	Canada	Nursing staff (N = 41)	Controlled clinical trial T0 = prior to intervention T1 = after the intervention T2 = 3 month follow-up	Self-efficacy Program, including didactic information and discussion and experiential role-playing	4 sessions of ~ 120 min over the course of 1 month	↑ Personal accomplishment − Depersonalisation − Emotional exhaustion − Satisfaction with teamwork	No statistically significant impact	Strong
O'Brien, W.H.	2019 ^c^	USA	Nurses and nursing aides (N = 71)	Cluster randomised controlled trial T0 = prior to intervention T1 = 1 month follow-up	Acceptance and Commitment Therapy (ACT)	3 sessions of ~ 150 min. spaced 1 week apart	↓ Mental health symptoms	Statistically significant positive impact	Strong
Schrijnemaekers, V.J.J.	2003	The Netherlands	Nursing staff (N = 300)	Randomised controlled trial T0 = prior to intervention T1 = 3 months follow-up T2 = 6 months follow-up T3 = 12 months follow-up	Clinical lessons, a training program and supervision meetings in emotion oriented care	2 lessons of ~ 60 min., 6 days training and 3 supervision meetings of ~ 240 min. over the course of 4 months	↑ Satisfaction with opportunities for self-actualisation ↑ Satisfaction with contact with residents (study specific) ↑ Job satisfaction ↑ Personal accomplishment − Satisfaction with contact with residents (general scale) − Satisfaction with good care for residents (study specific) − (one) General satisfaction item − Satisfaction with head of the ward − Satisfaction with quality of care − Satisfaction with contact with colleagues − Depersonalisation − Emotional exhaustion	No statistically significant impact	Strong
Yu, C.Y.	2012 ^b^	Taiwan	Nursing assistants (N = 83)	Controlled clinical trial T0 = prior to intervention T1 = 4 weeks follow-up	Elderly Simulation Program in which participants experience what it is like to have degenerating physical functioning (e.g. special glasses to simulate poor eyesight)	1 lecture of ~ 60 min. and 1 simulation of ~ 60 min	− Motivation to care for older adults	No statistically significant impact	Strong
Brazil, K.	1998	Canada	Registered (practical) nurses (N = 51)	Controlled clinical trial T0 = prior T1 = 2 months follow-up	A geriatric nursing education workshop	2 days	− Job satisfaction	No statistically significant impact	Moderate
Buruck, G.	2016 ^b^	Germany	Nursing staff (N = 96)	Randomised controlled trial T0 = prior to intervention T1 = after the intervention T2 = 6 months follow-up	An emotion regulation training (Affect Regulation Training (ART)), including cognitive behavioural therapy and skill building audio material	8 to 9 sessions of ~ 90 min. over the course of 8 to 12 weeks	↑ Well-being	Statistically significant positive impact	Moderate
Davison, T.E.	2007 ^b^	Australia	Registered nurses and nursing assistants (N = 90)	Randomised controlled trial T0 = 1 week prior to intervention T1 = after the intervention T2 = 6 months follow-up	A didactic and experiential learning program in managing dementia related challenging behaviours with a peer support group	8 sessions dementia training of 60–90 min. and 5 peer support sessions of 30–60 min	− Personal accomplishment − Depersonalisation − Emotional exhaustion	No statistically significant impact	Moderate
Kossek, E.E.	2019	USA	Staff (N = 1524)	Randomised controlled trial T0 = prior to intervention T1 = 6 months follow-up T2 = 12 months follow-up T3 = 18 months follow-up	A multi-level intervention integrating two work interventions, including employee group sessions, work-improvement redesign activities, leader computer-based training, and behavioural self-monitoring by leaders and co-workers	multiple sessions over the course of 4 months	− Perceived stress − Psychological distress	No statistically significant impact	Moderate
Moyle, W.	2013 ^c^	Australia	Nursing staff (N = 19)	Randomised controlled trial T0 = prior to intervention T1 = after the intervention	Foot massage intervention at work	Max 3 sessions a week of ~ 10 min. over the course of 4 weeks	↓ Anxiety − Satisfaction	Statistically significant positive impact	Moderate
Pillemer, K.	2003	USA	Nursing staff (N = 655)	Randomised controlled trial T0 = prior to intervention T1 = 8 weeks follow-up T2 = 6 months follow-up	Parallel workshops, one for staff and one for family members, about cooperation and effective communication between staff and family	1 session of ~ 420 min. and 1 joint session with both staff and family	− Depersonalisation	No statistically significant impact	Moderate
Engst, C.	2005 ^c^	Canada	Nursing staff (N = 50)	Controlled clinical trial T0 = prior to intervention T1 = 12 month follow-up	The instalment and use of overhead ceiling lifts including a 'no-unsafe manual lift' policy	1 session of ~ 60 min	− Staff satisfaction	No statistically significant impact	Weak
Franzmann, J.	2016 ^b^	Germany	Nursing staff (N = 116)	Controlled clinical trial T0 = prior to intervention T1 = after the intervention T2 = 6 months follow-up	A train-the-trainer in a program to foster communication skills attending the needs of people with dementia	Every 2 weeks sessions of ~ 120 min. over the course of 6 months	↓ Occupational mental stress	Statistically significant positive impact	Weak
Visser, S.M.	2006	Australia	Staff (N = 52)	Randomised controlled trial T0 = prior to intervention T1 = after the intervention T2 = 3 month follow-up T3 = 6 month follow-up	Education programme with a peer support group to manage behavioural symptoms of dementia	2 session of ± 1.5 h with additional 30 min. peer support training a week over the course of 4 weeks	− Personal accomplishment − Depersonalisation − Emotional exhaustion	No statistically significant impact	Weak
Zwijsen, S.A.	2014	The Netherlands	Nursing staff (N = 380)	Stepped wedge cluster randomised controlled trial T0 = prior to intervention T1 = midway through the implementation process T2 = after the intervention	Care programme, adapted to the education level of users, for challenging behaviour, including education, multidisciplinary consultation and several forms	Ongoing process of detection, analysis, treatment, and evaluation of challenging behaviour in a multidisciplinary setting over the course of 1.5 year	↑ Job satisfaction − Personal accomplishment − Depersonalisation − Emotional exhaustion	No statistically significant impact	Weak

Study outcomes related to employability									
Broughton, M.	2011 ^a^	Australia	Nursing assistants, registered/endorsed/enrolled nurses and recreational/activities officers (N = 68)	Controlled clinical trial T0 = prior to intervention T1 = after the intervention T2 = 3 months follow-up	A DVD-based caregiver education program to support memory and communication in dementia, including a booklet summarizing information	1 session of ~ 90 min	↑ Knowledge of strategies to support memory and communication in dementia	Statistically significant positive impact	Strong
Chang, C.	2005	Taiwan	Nursing assistants (N = 67)	Randomised controlled trial T0 = prior to intervention T1 = after the intervention T2 = 4 weeks follow-up	A feeding skills training program, including in-service classes and hands-on training	2 days	↑ Knowledge of feeding dementia patients	Statistically significant positive impact	Strong
Kuske, B.	2009 ^a,^^c^	Germany	Nursing home staff (N = 134)	Cluster randomised controlled trial T0 = prior to intervention T1 = after the intervention T2 = 6 months follow-up	Training in dealing and interacting with people with dementia, including lectures, videotapes, handouts, brainstorming and short games	13 weekly sessions of ~ 60 min. over the course of 13 weeks	↑ Knowledge in dealing with challenging problem behaviours of people with dementia ↑ Expertise in dealing with challenging problem behaviours of people with dementia ↑ Overall competence ↑ Social competence − Methodological competence − Personal competence − Competence in responding to colleagues	Statistically significant positive impact	Strong
Mackenzie, C.S.	2003 ^a^	Canada	Nursing staff (N = 41)	Controlled clinical trial T0 = prior to intervention T1 = after the intervention T2 = 3 month follow-up	Self-efficacy Program, including didactic information and discussion and experiential role-playing	4 sessions of ~ 120 min over the course of 1 month	↑ Self-efficacy − Knowledge regarding ways of improving teamwork and coping with challenging residents' and their families	Statistically significant positive impact	Strong
Yu, C.Y.	2012 ^a^	Taiwan	Nursing assistants (N = 83)	Controlled clinical trial T0 = prior to intervention T1 = 4 weeks follow-up	Elderly Simulation Program in which participants experience what it is like to have degenerating physical functioning (e.g. special glasses to simulate poor eyesight)	1 lecture of ~ 60 min. and 1 simulation of ~ 60 min	↑ Knowledge about aging	Statistically significant positive impact	Strong
Buruck, G.	2016 ^a^	Germany	Nursing staff (N = 96)	Randomised controlled trial T0 = prior to intervention T1 = after the intervention T2 = 6 months follow-up	An emotion regulation training (Affect Regulation Training (ART)), including cognitive behavioural therapy and skill building audio material	8 to 9 sessions of ~ 90 min. over the course of 8 to 12 weeks	− Emotion regulation skills	No statistically significant impact	Moderate
Davison, T.E.	2007 ^a^	Australia	Registered nurses and nursing assistants (N = 90)	Randomised controlled trial T0 = 1 week prior to intervention T1 = after the intervention T2 = 6 months follow-up	A didactic and experiential learning program in managing dementia related challenging behaviours with a peer support group	8 sessions dementia training of 60–90 min. and 5 peer support sessions of 30-60 min	↑ Self-efficacy	Statistically significant positive impact	Moderate
Janssens, B.	2017	Belgium	Nurses and nursing aides (N = 2409)	Controlled clinical trial T0 = prior to intervention T1 = after the intervention (approx. 16 months)	An oral health care programme, including the implementation of an oral healthcare team, education, the implementation of a guideline and regular visits of a mobile dental team	Implementation of the program over the course of ~ 21 months in which a mobile dental team delivered care for ~ 11 days a year	↑ Knowledge of oral health and – hygiene	Statistically significant positive impact	Moderate
Janssens, B.	2014	Belgium/Netherlands	Nurses and nursing aides (N = 760)	Cluster randomised controlled trial T0 = prior to intervention T1 = 6 months follow-up	An oral health care programme, including education, the implementation of a guideline and setting up an oral health care record for each resident	1.5 h lecture by investigator, 2 h lecture and 1 h practical education of the oral health care team, 1.5 h session at each ward over the course of 1 week. The implementation of the guideline over the course of 6 months	↑ Knowledge of oral health and – hygiene	Statistically significant positive impact	Moderate
Mellor, D.	2010	Australia	Direct carers, care managers, personal care assistants, registered nurses (N = 244)	Randomised controlled trial T0 = prior to intervention T1 = after the intervention T2 = 3 months follow-up	Depression Training Program, including basic information and additional sessions for registered nurses and care managers	4 sessions of ~ 90 min. and 2 additional sessions of ~ 120 min. sessions over the course of 6 weeks	↑ Direct carers and personal care assistants' knowledge of depression ↑ Direct carers and personal care assistants' self-efficacy ↑ Care managers and registered nurses' self-efficacy − Care managers and registered nurses' knowledge of depression	Statistically significant positive impact	Moderate
Pellfolk, T.J.E.	2010	Sweden	Nursing staff (N = 393)	Cluster randomised controlled trial T0 = prior to intervention T1 = 6 months follow-up	Restraint-minimisation education, including videotaped lectures for all and a seminar for one volunteer of each unit	6 videotaped lectures of 30 min. over the course of 6 months	↑ Knowledge of dementia care and laws	Statistically significant positive impact	Moderate
Bourgeois, M.S.	2004	USA	Nursing aides (N = 126)	Pre test – repeated posttest control group design T0 = prior to intervention T1 = after the intervention T2 = 3 month follow-up	A multicomponent communication skills training, including didactive in-service training, one-on-one training, use of memory books, self-monitoring and supervisory feedback	~ 8 sessions of 60 min. over the course of 2–3 weeks	↑ Effective communication skills ↑ Effective instruction giving − Ineffective instruction giving − Knowledge about communicating effectively	Statistically significant positive impact	Weak
Conway, E.R.	2016	Australia	Community-based aged care staff (N = 59)	Cluster randomised controlled trial T0 = prior to intervention T1 = after the intervention T2 = 3 months follow-up	A DVD-based caregiver education program to support communication in dementia, including a booklet summarizing information (resembling Broughton, 2011)	1 session of ~ 60 min. and individual feedback at 1, 2 and 6 weeks post training	− Knowledge of communication strategies in dementia − Self-efficacy	No statistically significant impact	Weak
Ersek, M.	2005	USA	Nursing staff (N = 169)	Controlled clinical trial T0 = prior to intervention T1 = 4 weeks follow-up T2 = 6 months follow-up	An educational end-of-life program, including newsletters and reminders	1 day every month over the course of 4 months	↑ End-of-life knowledge ↑ Confidence in end-of-life skills ↑ Performance of end-of-life skills (rated by supervisors)	Statistically significant positive impact	Weak
Franzmann, J.	2016 ^a^	Germany	Nursing staff (N = 116)	Controlled clinical trial T0 = prior to intervention T1 = after the intervention T2 = 6 months follow-up	A train-the-trainer in a program to foster communication skills attending the needs of people with dementia	Every 2 weeks sessions of ~ 120 min. over the course of 6 months	↑ Social competence	Statistically significant positive impact	Weak
Kong, E.	2017	Korea	Nursing staff (N = 126)	Cluster randomised controlled trial T0 = prior to intervention T1 = after the intervention T2 = 1 month follow-up T3 = 3 months follow-up	A multicomponent Restraint Reduction Program, including three educational session (two classroom and one self-directed web based education) and two unit based consultations	~ 135 min. education and ~ 120 min. consultation over the course of 6 weeks	↑ Knowledge about physical restraint	Statistically significant positive impact	Weak
Van Gaal, B.G.I.	2010	The Netherlands	Nurses (N = 558)	Cluster randomised controlled trial T0 = prior to intervention T1 = 12 month follow-up	Educational patient safety knowledge programme	~ 2–3 educational sessions of ~ 90 min., 2 case discussions of 30 min., and 2 outreach visits of ~ 300 min over the course of several months	↑ Hospital staff’s knowledge regarding pressure ulcers − Hospital staff’s knowledge regarding; urinary tract infections and falls − Nursing home staff’s knowledge regarding; pressure ulcers, urinary tract infections and falls	No statistically significant impact	Weak

^a^This study also measured outcomes related to vitality

^b^This study also measured outcomes related to employability

^c^This study also measured outcomes related to workability

Outcomes are shown to increases (↑), decreases (↓) or ( −) non-significant (p > .05) between groups over time

### Quality Assessment

The overall methodological quality of the studies varied from weak, to moderate and strong (Table 3). Twelve studies were rated as strong, twelve as moderate and nine as weak. Since our review only included studies with a control group, all studies were designed either as a randomised controlled trial or a controlled trial (without randomisation) and were therefore rated ‘strong’ with respect to the quality of their study design. All studies received a moderate rating on the category blinding, because most studies did not provide information on the outcome assessor(s) and participants’ awareness of relevant blinding issues (e.g. exposure status of the participant or the awareness of the research question). Indistinct reporting on withdrawals, dropouts, and validity and reliability of data collection tools, was for most studies reason for their overall weak quality rating.

**Table 3:** Quality assessment of included studies

First author	Publication year	Selection bias	Study design	Confounders	Blinding	Data collection methods	Withdrawals and drop-outs	Overall quality
Barbosa, A.	2015	Strong	Strong	Strong	Moderate	Strong	Strong	STRONG
Barbosa, A.	2016	Strong	Strong	Strong	Moderate	Strong	Strong	STRONG
Broughton, M.	2011	Moderate	Strong	Strong	Moderate	Strong	Moderate	STRONG
Chang, C.	2005	Moderate	Strong	Strong	Moderate	Strong	Strong	STRONG
Fukuda, K.	2018	Moderate	Strong	Strong	Moderate	Strong	Strong	STRONG
Kloos, N.	2019	Strong	Strong	Strong	Moderate	Strong	Moderate	STRONG
Kuske, B.	2009	Moderate	Strong	Strong	Moderate	Strong	Strong	STRONG
Mackenzie, C.S.	2003	Strong	Strong	Strong	Moderate	Strong	Moderate	STRONG
O'Brien, W.H.	2019	Moderate	Strong	Strong	Moderate	Strong	Moderate	STRONG
Rasmussen, C. D. N. R.	2015	Moderate	Strong	Strong	Moderate	Moderate	Moderate	STRONG
Schrijnemaekers, V. J. J.	2003	Strong	Strong	Strong	Moderate	Strong	Strong	STRONG
Yu, C. Y.	2012	Moderate	Strong	Strong	Moderate	Strong	Strong	STRONG
Brazil, K.	1998	Moderate	Strong	Weak	Moderate	Strong	Strong	MODERATE
Buruck, G.	2016	Moderate	Strong	Strong	Moderate	Strong	Weak	MODERATE
Davison, T.E.	2007	Moderate	Strong	Strong	Moderate	Strong	Weak	MODERATE
Dulon, M.	2009	Moderate	Strong	Strong	Moderate	Strong	Weak	MODERATE
Janssens, B.	2014	Strong	Strong	Weak	Moderate	Moderate	Moderate	MODERATE
Janssens, B.	2017	Moderate	Strong	Strong	Moderate	Moderate	Weak	MODERATE
Kossek, E.	2019	Moderate	Strong	Strong	Moderate	Strong	Weak	MODERATE
Mellor, D.	2010	Moderate	Strong	Weak	Moderate	Strong	Moderate	MODERATE
Moyle, W.	2013	Moderate	Strong	Strong	Moderate	Weak	Strong	MODERATE
Pellfolk, T.J.	2010	Strong	Strong	Strong	Moderate	Weak	Moderate	MODERATE
Pillemer, K.	2003	Strong	Strong	Strong	Moderate	Weak	Moderate	MODERATE
Skargren, E.	1996	Strong	Strong	Strong	Moderate	Weak	Moderate	MODERATE
Bourgeois, M. S.	2004	Moderate	Strong	Weak	Moderate	Weak	Weak	WEAK
Conway, E.R.	2016	Moderate	Strong	Strong	Moderate	Weak	Weak	WEAK
Engst, C.	2005	Moderate	Strong	Weak	Moderate	Weak	Weak	WEAK
Ersek, M.	2005	Moderate	Strong	Strong	Moderate	Weak	Weak	WEAK
Franzmann, J.	2016	Moderate	Strong	Weak	Moderate	Weak	Weak	WEAK
Kong, E.	2017	Moderate	Strong	Weak	Moderate	Weak	Strong	WEAK
Van Gaal, B. G. I.	2010	Moderate	Strong	Strong	Moderate	Weak	Weak	WEAK
Visser, S.M.	2006	Moderate	Strong	Weak	Moderate	Weak	Weak	WEAK
Zwijsen, S. A.	2014	Weak	Strong	Strong	Moderate	Strong	Weak	WEAK

### Effectiveness on Workability Outcomes

Seven studies reported about outcomes related to workability, such as skin symptoms, lower back pain or global physical impairment. Five out of seven interventions had a statistically significant positive impact on outcomes related to workability, meaning that within these five studies 50 percent or more of the outcome measures showed a statistically significant positive impact (p < 0.05). Those were interventions with a focus on employees’ physical health, namely a skin care program [38], the use of overhead ceiling lift program to prevent injury [39], a foot massage to improve blood pressure [40], an exercise program to improve physical health [41], and a multifaceted intervention including participatory ergonomics, cognitive behaviour training and physical training to prevent lower back pain [42]. The two interventions showing no statistically significant impact were mainly focused on improving employees’ skills, and encompassed a training in dementia care [43] and an acceptance and commitment therapy intervention [44].

When taking all the studies into account, less than 75 percent of the studies (5 out of 7, 71%) showed statistically significant results in the same (positive) direction, resulting in an overall rating of insufficient evidence. The sensitivity analysis, only including interventions directly addressing physical health and functional capacities of employees, revealed that all studies (5 out of 5, 100%) showed a statistically significant positive impact in multiple strong/moderate quality studies. According to the sensitivity analysis there is strong level of evidence for the effectiveness of interventions aimed at improving outcomes related to workability of aged care staff.

### Effectiveness on Vitality Outcomes

More than half of the included studies in this review (19 of the 32), reported about outcomes related to vitality. Levels of burnout (i.e. emotional exhaustion, personal accomplishment and depersonalisation) and satisfaction were amongst the most frequently assessed outcomes, and were incorporated in fourteen studies. Only four studies showed a statistically significant positive impact on outcomes related to vitality, meaning that within these four studies 50 percent or more of the outcome measures showed a statistically significant positive impact (p < 0.05). One of them was a relaxing foot massage [40] and the other three were interventions targeting skills of the employee, i.e., an emotion regulation training [45], an acceptance and commitment therapy intervention [44], and a train-the-trainer in dementia care intervention [46]. The interventions that showed no statistically significant impact on increasing vitality were interventions focusing on education about person-centred care, geriatric nursing, communication, challenging behaviours, cooperation and emotion oriented care [36, 37, 47–53]. Other interventions with no statistically significant impact focused on skills (positive psychology, dealing with people with dementia, self-efficacy, cooperation and communication or experiencing degenerating physical functioning) or included a change in the workplace (instalment of ceiling lifts and job redesign) [39, 43, 54–58]. As less than 75 percent of the studies (4 out of 19, 21%) showed statistically significant results in the same direction, the level of evidence for vitality is rated as insufficient. For the sensitivity analysis we included three studies directly addressing the energy and motivation of employees themselves, which where the emotion regulation training, the positive psychology intervention and the acceptance and commitment therapy [44, 45, 54]. The sensitivity analysis revealed that 2 out of 3 studies (67%) showed a statistically significant impact, whereby indicating that the level of evidence for the effectiveness of interventions aimed at improving vitality of aged care staff is insufficient.

### Effectiveness on Employability Outcomes

Over half of the included studies in this review (17 out of 32) reported about outcomes related to employability. Knowledge is the most frequently reported outcome regarding this component, and is measured in fourteen studies. Fourteen interventions showed a statistically significant positive impact on outcomes related to employability, meaning that within these fourteen studies 50 percent or more of the outcome measures showed a statistically significant positive impact (p < 0.05). These interventions all focused on improving knowledge and/or skills of the employee (e.g. oral health care, restraint reduction, communication, dealing with challenging behaviour, self-efficacy) [43, 46, 48, 49, 56, 58–66].

Since more than 75 percent of the studies (14 out of 17, 82%) showed statistically significant results in the same (positive) direction and multiple were of high quality (5 strong and 5 moderate quality studies), the level of evidence is rated as strong. Since all studies had a clear link to the competence and skills of employees, no additional sensitivity analysis was conducted.

## Discussion

This review aimed to investigate the effectiveness of workplace interventions at team- or organisational workplace level on each of the three components (i.e,. workability, vitality, employability) of sustainable employability of healthcare professionals caring for older adults. We found strong level of evidence for effects of workplace interventions on employability and for workplace interventions directly targeting workability. Evidence for effects of interventions on vitality was insufficient.

Regarding workability, it is remarkable that we did not find strong evidence for the effectiveness of interventions when taking all interventions into account. However, we did find strong level of evidence when only taking into account interventions directly addressing workability. All interventions with a statistically significant positive impact had a direct link to the core of our operationalisation of workability, namely the physical health of employees. In contrast, interventions that did not have a direct link to physical health of the employee (i.e. dementia care and acceptance and commitment therapy) did not report a statistically significant impact on workability. The reviews by Oakman et al. [22] and Cloostermans et al. [21] also found no effect of interventions targeting behaviour change through education on workability. In contrast to our findings, they also did not find an impact of physical activity interventions on workability. Since our review included indicators of workability (and not workability itself), the effects in our review were possibly more direct and short-term effects of the interventions. Future research should disentangle whether these effects on indicators of workability also have a significant and enduring impact over time.

The results concerning outcomes related to vitality show a more complicated picture. Very few studies had a statistically significant impact on outcomes related to the vitality of employees. The interventions with statistically significant impact varied in terms of approach, content and outcome measures. One of them was a relaxing foot massage [40] and the other three were interventions in which very different kinds of skills of the employee were targeted, such as emotion regulation [45], acceptance and commitment/mindfulness [44], and skills to train others in dementia care [46]. The level of evidence for the effectiveness of interventions aimed at improving vitality of healthcare professionals in aged care was rated insufficient. This result is in line with the return-to-work literature showing insufficient and mixed evidence for workplace interventions on outcomes related to quality-of-life (e.g. mental health) [67]. What stands out in our review is that almost all studies on vitality without statistically significant impact were primarily aimed at improving the workplace or knowledge/skills of the employee and expected outcomes related to vitality to be the secondary/indirect effect of the intervention [36, 37, 39, 43, 47–53, 55, 57, 58]. To illustrate, an educational intervention can be effective in improving skills (employability), but rarely has an additional impact on employee well-being or stress (vitality) [46, 48]. It could be that interventions aimed at vitality are more often focused on the individual and therefore not included in this review. Nonetheless, our finding is congruent with the review of Hazelzet et al. [23] who suggested that the misalignment of the outcome measures and intervention content explains the limited evidence for effectiveness of the interventions. In order to attain effects of workplace interventions at the vitality of employees it thus seems important that the interventions focuses on that component specifically. To draw conclusions about the effectiveness of interventions on outcomes of vitality, future research should dive into the success factors of the few successful current interventions. Effect- and process evaluations of new workplace interventions aiming to improve the energy and motivation of staff should provide insight into the effectiveness of these vitality-directed interventions.

Our finding of strong level of evidence for the effectiveness of interventions on outcomes of employability is in line with the review of Hazelzet et al. [23] showing that interventions can have positive effects when aimed at having the right competences to perform the job, and development of skills and knowledge. In our review we found that almost all interventions focused on improving knowledge and/or skills of employees showed statistically significant impact in doing so, which is in agreement with the predominantly effective educational initiatives in several topics in nursing care [68, 69].

### Methodological Considerations

For our literature search we used a very thorough search strategy that was developed with an experienced librarian. On the one hand we searched for well-designed studies (with both pre-post measures and a control group) in a specific target population (healthcare professionals caring for older adults) with broad outcome measures (related to sustainable employability). Our search therefore resulted in a rich sample of interventions, in which we exclusively focused on outcomes that we considered indicative of sustainable employability according to the classification as described by De Vos and Van der Heijden [20]. The decision to consider a certain outcome as an indicator of workability, vitality or employability was to some extent subjective, since there is no consensus among scholars about the definition and components of sustainable employability [20, 70].

On the other hand, our search strategy led to heterogeneity in outcomes related to a component of sustainable employability. As a result, specific information about the methods and effects of single studies are lost in general statements about the effectiveness of interventions on the level of the components of sustainable employability. For example, we did not differentiate between studies with long and short-term follow-up, online versus offline interventions, or interventions including one or multiple sessions. Since the review focused on effect evaluations, and not on process evaluations, it is unknown whether the lack of evidence for components of sustainable employability were either due to program failure or theory failure [71].

The diversity of the sample is also visible in the quality assessments. The quality of the included articles varied from weak, to moderate, to strong. In order to be rated as ‘strong’ on the data collection component of the quality assessment tool, studies had to provide information about the validity of the measures that were incorporated. Not all articles included this information, resulting in an immediate weak score on this component.

To assess the level of evidence, we have postulated a sensitivity analysis post-hoc, because we noted during data extraction that the alignment between the intervention and outcome measures differed between studies. This additional analysis enabled us to show that the link between the intervention and outcome is an important factor in the level of evidence for effectiveness of the interventions.

The overall level of evidence rating scheme is based on the previously used scheme by Hoogendoorn et al. [34] and Van Drongelen et al. [35], which involves the counting of effective studies versus ineffective studies based on their attained outcome measures’ *p*-value. With this method a nonsignificant finding could be due to a true absence of the effect, but it can also be due to low statistical power [72]. Also, *p*-values do not state anything about the size or clinical relevance of effects, since statistical significance does not necessarily imply large or meaningful effects [73]. The used method therefore provides more insight into the level of evidence for effectiveness of interventions on components of sustainable employability, rather than referring to the size or clinical relevance of the findings. Effect sizes that were reported in the full-texts of the studies varied from small, to medium and large [40, 43–45, 48].

### Practical Implications and Recommendations for Future Research

As a result of this review, we have a number of recommendations for facilities providing care for older adults, if they wish to improve employees’ sustainable employability. Firstly, we would advise these facilities to prioritize one component of sustainable employability within their organisation, because to date we have no (evaluated) interventions that show statistically significant positive effects on all three of its components. Secondly, it is important that organisations look for an intervention that matches the component they intend to improve. For workability this means that interventions should focus on the physical health and functional capacities of employees and for employability the main focus should lie on development of knowledge and skills. Our review does not offer much evidence for the effectiveness of workplace vitality-directed interventions. Therefore, future research should focus on evaluation of interventions aimed at improving the energy and motivation of employees at team or organisational level as this will enable us to draw conclusions about the effectiveness of such interventions on vitality.

## Concluding Remarks

We found different levels of evidence for workplace interventions on sustainable employability of aged care staff. We found a strong level of evidence for—small to large sized—effects of workplace interventions on employability and for workplace interventions directly targeting workability. For workability this means that interventions should focus at the physical health and functional capacities of employees and for employability the main focus should lie on the development of knowledge and skills. Evidence for effects of interventions on vitality was insufficient. More research is needed towards workplace interventions aimed at improving vitality to draw conclusions about the effectiveness of such interventions on vitality. This is necessary because of the focus of workplace interventions to the component of the sustainable employability it is aiming to improve, is important for its effectiveness.

## Supplementary Information

Below is the link to the electronic supplementary material.

Supplementary file1—Search Strategy (DOCX 18 kb)

Supplementary file2—PRISMA 2020 checklist (DOCX 42 kb)

## Data Availability

All data generated or analysed during this study are included in this published article and its supplementary information files.
