# Supplementary material for: Effectiveness of Team and Organisational Level Workplace Interventions Aimed at Improving Sustainable Employability of Aged Care Staff: A Systematic Review
Source: J Occup Rehabil. 2022 Sep 23;33(1):37–60. doi: 10.1007/s10926-022-10064-5 (PMC10025231; doi:10.1007/s10926-022-10064-5)
Supplement: Supplementary file 1 — Supplementary file1—Search Strategy (DOCX 18 kb) [file 10926_2022_10064_MOESM1_ESM.docx]

**Effectiveness of Team and Organisational Workplace Interventions Aimed at Improving Sustainable Employability of Aged Care Staff: A Systematic Review**

Ceciel H. Heijkants, MSc.^1^, Dr. Astrid de Wind^2^, Dr. Madelon L. M. van Hooff^1^, Prof. Dr. Sabine A. E. Geurts^1^, and Prof. Dr. Cécile R. L. Boot^1,3^

^1^ Radboud University, Behavioural Science Institute, The Netherlands

^2^ Amsterdam UMC, University of Amsterdam, Department of Public and Occupational Health, Coronel Institute of Occupational Health, Amsterdam Public Health research institute, The Netherlands

^3^ Amsterdam UMC, VU University, Amsterdam Public Health research institute, department of Public and Occupational Health, The Netherlands

**Author Note**

We have no known conflict of interest to disclose.

This study is funded by the Foundation Joannes de Deo, 24001506 (ID 243207).

We would like to acknowledge the librarians of the Radboud University who helped developing our search strategy.

Correspondence concerning this article should be addressed to Ceciel Heijkants, Thomas van Aquinostraat 4, 6525GD Nijmegen, room 04.362. Email: [Ceciel.Heijkants@ru.nl](mailto:Ceciel.Heijkants@ru.nl)

**Supplementary material – Search Strategy**

| **Search strings** | **Embase** | **PsycINFO** | **MEDLINE** | **Web of science** | **CINAHL** |
| --- | --- | --- | --- | --- | --- |
| 1 | ("care worker*" or "careworker*" or "care giver*" or "caregiver*" or "care provider*" or "careprovider*" or "nurse" or "nurses").ab,ti. | ("care worker*" or "careworker*" or "care giver*" or "caregiver*" or "care provider*" or "careprovider*" or "nurse" or "nurses").ab,ti. | ("care worker*" or "careworker*" or "care giver*" or "caregiver*" or "care provider*" or "careprovider*" or "nurse" or "nurses").ab,ti. | TS=(“care worker*” or “careworker*” or “care giver*” or “caregiver*” or “care provider*” or “careprovider*” or “nurse” or “nurses”)  *Indexes=SCI-EXPANDED, SSCI, A&HCI, ESCI Timespan=All years* | ( AB (“care worker*” or “careworker*” or “care giver*” or “caregiver*” or “care provider*” or “careprovider*” or “nurse” or “nurses”) ) OR ( TI (“care worker*” or “careworker*” or “care giver*” or “caregiver*” or “care provider*” or “careprovider*” or “nurse” or “nurses”) ) |
| 2 | ("personnel" or "staff" or "work force" or "workforce" or "workplace" or "organi?ation*" or "job").ab,ti. | ("personnel" or "staff" or "work force" or "workforce" or "workplace" or "organi?ation*" or "job").ab,ti. | ("personnel" or "staff" or "work force" or "workforce" or "workplace" or "organi?ation*" or "job").ab,ti. | TS=(“personnel” or “staff” or “work force” or “workforce” or “workplace” or “organi?ation*” or “job”)  *Indexes=SCI-EXPANDED, SSCI, A&HCI, ESCI Timespan=All years* | ( AB (“personnel” or “staff” or “work force” or “workforce” or “workplace” or “organi?ation*” or “job”) ) OR ( TI (“personnel” or “staff” or “work force” or “workforce” or “workplace” or “organi?ation*” or “job”) ) |
| 3 | ("extended care facilit*" or "geriatric*" or "elder* care" or "long term care home*" or "long term care facilit*" or "long term care institution*" or "long term care setting*" or "long term care residen*" or "long-term care home*" or "long-term care facilit*" or "long-term care institution*" or "long-term care setting*" or "long-term care residen*" or "longterm care home*" or "longterm care facilit*" or "longterm care institution*" or "longterm care setting*" or "longterm care residen*" or "LTC home*" or "LTC facilit*" or "LTC institution*" or "LTC setting*" or "LTC residen*" or "LTC provider*" or "residential home*" or "residential care" or "residential facilit*" or "residential institution*" or "residential setting*" or "long stay home*" or "long stay facilit*" or "long stay institution*" or "long stay setting*" or "long stay residen*" or "long-stay home*" or "long-stay facilit*" or "long-stay institution*" or "long-stay setting*" or "long-stay residen*" or "longstay home*" or "longstay facilit*" or "longstay institution*" or "longstay setting*" or "longstay residen*" or "nursing home*" or "nursing facilit*" or "nursing institution*" or "nursing setting*" or "institutionali*" or "institutional care" or "LTCF" or "care home*" or "care facilit*" or "care institution*" or "care setting*" or "care residen*" or "rest home*" or "aged care" or "dementia care" or "homes for the aged" or "skilled nursing facilit*" or "assisted living").ab,ti. | ("extended care facilit*" or "geriatric*" or "elder* care" or "long term care home*" or "long term care facilit*" or "long term care institution*" or "long term care setting*" or "long term care residen*" or "long-term care home*" or "long-term care facilit*" or "long-term care institution*" or "long-term care setting*" or "long-term care residen*" or "longterm care home*" or "longterm care facilit*" or "longterm care institution*" or "longterm care setting*" or "longterm care residen*" or "LTC home*" or "LTC facilit*" or "LTC institution*" or "LTC setting*" or "LTC residen*" or "LTC provider*" or "residential home*" or "residential care" or "residential facilit*" or "residential institution*" or "residential setting*" or "long stay home*" or "long stay facilit*" or "long stay institution*" or "long stay setting*" or "long stay residen*" or "long-stay home*" or "long-stay facilit*" or "long-stay institution*" or "long-stay setting*" or "long-stay residen*" or "longstay home*" or "longstay facilit*" or "longstay institution*" or "longstay setting*" or "longstay residen*" or "nursing home*" or "nursing facilit*" or "nursing institution*" or "nursing setting*" or "institutionali*" or "institutional care" or "LTCF" or "care home*" or "care facilit*" or "care institution*" or "care setting*" or "care residen*" or "rest home*" or "aged care" or "dementia care" or "homes for the aged" or "skilled nursing facilit*" or "assisted living").ab,ti. | ("extended care facilit*" or "geriatric*" or "elder* care" or "long term care home*" or "long term care facilit*" or "long term care institution*" or "long term care setting*" or "long term care residen*" or "long-term care home*" or "long-term care facilit*" or "long-term care institution*" or "long-term care setting*" or "long-term care residen*" or "longterm care home*" or "longterm care facilit*" or "longterm care institution*" or "longterm care setting*" or "longterm care residen*" or "LTC home*" or "LTC facilit*" or "LTC institution*" or "LTC setting*" or "LTC residen*" or "LTC provider*" or "residential home*" or "residential care" or "residential facilit*" or "residential institution*" or "residential setting*" or "long stay home*" or "long stay facilit*" or "long stay institution*" or "long stay setting*" or "long stay residen*" or "long-stay home*" or "long-stay facilit*" or "long-stay institution*" or "long-stay setting*" or "long-stay residen*" or "longstay home*" or "longstay facilit*" or "longstay institution*" or "longstay setting*" or "longstay residen*" or "nursing home*" or "nursing facilit*" or "nursing institution*" or "nursing setting*" or "institutionali*" or "institutional care" or "LTCF" or "care home*" or "care facilit*" or "care institution*" or "care setting*" or "care residen*" or "rest home*" or "aged care" or "dementia care" or "homes for the aged" or "skilled nursing facilit*" or "assisted living").ab,ti. | TS=(“extended care facilit*” or “geriatric*” or “elder* care” or “long term care home*” or “long term care facilit*” or “long term care institution*” or “long term care setting*” or “long term care residen*” or “long-term care home*” or “long-term care facilit*” or “long-term care institution*” or “long-term care setting*” or “long-term care residen*” or “longterm care home*” or “longterm care facilit*” or “longterm care institution*” or “longterm care setting*” or “longterm care residen*” or “LTC home*” or “LTC facilit*” or “LTC institution*” or “LTC setting*” or “LTC residen*” or “LTC provider*” or “residential home*” or “residential care” or “residential facilit*” or “residential institution*” or “residential setting*” or “long stay home*” or “long stay facilit*” or “long stay institution*” or “long stay setting*” or “long stay residen*” or “long-stay home*” or “long-stay facilit*” or “long-stay institution*” or “long-stay setting*” or “long-stay residen*” or “longstay home*” or “longstay facilit*” or “longstay institution*” or “longstay setting*” or “longstay residen*” or “nursing home*” or “nursing facilit*” or “nursing institution*” or “nursing setting*” or “institutionali*” or “institutional care” or “LTCF” or “care home*” or “care facilit*” or “care institution*” or “care setting*” or “care residen*” or “rest home*” or “aged care” or “dementia care” or “homes for the aged” or “skilled nursing facilit*” or “assisted living”)  *Indexes=SCI-EXPANDED, SSCI, A&HCI, ESCI Timespan=All years* | ( AB (“extended care facilit*” or “geriatric*” or “elder* care” or “long term care home*” or “long term care facilit*” or “long term care institution*” or “long term care setting*” or “long term care residen*” or “long-term care home*” or “long-term care facilit*” or “long-term care institution*” or “long-term care setting*” or “long-term care residen*” or “longterm care home*” or “longterm care facilit*” or “longterm care institution*” or “longterm care setting*” or “longterm care residen*” or “LTC home*” or “LTC facilit*” or “LTC institution*” or “LTC setting*” or “LTC residen*” or “LTC provider*” or “residential home*” or “residential care” or “residential facilit*” or “residential institution*” or “residential setting*” or “long stay home*” or “long stay facilit*” or “long stay institution*” or “long stay setting*” or “long stay residen*” or “long-stay home*” or “long-stay facilit*” or “long-stay institution*” or “long-stay setting*” or “long-stay residen*” or “longstay home*” or “longstay facilit*” or “longstay institution*” or “longstay setting*” or “longstay residen*” or “nursing home*” or “nursing facilit*” or “nursing institution*” or “nursing setting*” or “institutionali*” or “institutional care” or “LTCF” or “care home*” or “care facilit*” or “care institution*” or “care setting*” or “care residen*” or “rest home*” or “aged care” or “dementia care” or “homes for the aged” or “skilled nursing facilit*” or “assisted living”) ) OR ( TI (“extended care facilit*” or “geriatric*” or “elder* care” or “long term care home*” or “long term care facilit*” or “long term care institution*” or “long term care setting*” or “long term care residen*” or “long-term care home*” or “long-term care facilit*” or “long-term care institution*” or “long-term care setting*” or “long-term care residen*” or “longterm care home*” or “longterm care facilit*” or “longterm care institution*” or “longterm care setting*” or “longterm care residen*” or “LTC home*” or “LTC facilit*” or “LTC institution*” or “LTC setting*” or “LTC residen*” or “LTC provider*” or “residential home*” or “residential care” or “residential facilit*” or “residential institution*” or “residential setting*” or “long stay home*” or “long stay facilit*” or “long stay institution*” or “long stay setting*” or “long stay residen*” or “long-stay home*” or “long-stay facilit*” or “long-stay institution*” or “long-stay setting*” or “long-stay residen*” or “longstay home*” or “longstay facilit*” or “longstay institution*” or “longstay setting*” or “longstay residen*” or “nursing home*” or “nursing facilit*” or “nursing institution*” or “nursing setting*” or “institutionali*” or “institutional care” or “LTCF” or “care home*” or “care facilit*” or “care institution*” or “care setting*” or “care residen*” or “rest home*” or “aged care” or “dementia care” or “homes for the aged” or “skilled nursing facilit*” or “assisted living”) ) |
| 4 | ("intervention" or "training" or "program" or "programme").ab,ti. | ("intervention" or "training" or "program" or "programme").ab,ti. | ("intervention" or "training" or "program" or "programme").ab,ti. | TS=(“intervention” or “training” or “program” or “programme”)  *Indexes=SCI-EXPANDED, SSCI, A&HCI, ESCI Timespan=All years* | ( AB ("intervention" or "training" or "program" or "programme") ) OR ( TI ("intervention" or "training" or "program" or "programme") ) |
| 5 | ("control* stud*" or "clinical trial*" or "random* control* trial*" or "cluster random* trial*" or "case control stud*" or "pre test posttest control group design" or "pretest" or "posttest" or "static group comparison" or "pilot stud*" or "quasiexperiment*" or "quasi experiment*" or "evaluat*" or "time series" or "time point*" or "repeated measur*").tw. | ("control* stud*" or "clinical trial*" or "random* control* trial*" or "cluster random* trial*" or "case control stud*" or "pre test posttest control group design" or "pretest" or "posttest" or "static group comparison" or "pilot stud*" or "quasiexperiment*" or "quasi experiment*" or "evaluat*" or "time series" or "time point*" or "repeated measur*").tw. | ("control* stud*" or "clinical trial*" or "random* control* trial*" or "cluster random* trial*" or "case control stud*" or "pre test posttest control group design" or "pretest" or "posttest" or "static group comparison" or "pilot stud*" or "quasiexperiment*" or "quasi experiment*" or "evaluat*" or "time series" or "time point*" or "repeated measur*").tw. | ALL=(“control* stud*” or “clinical trial*” or “random* control* trial*” or “cluster random* trial*” or “case control stud*” or “pre test posttest control group design” or “pretest” or “posttest” or “static group comparison” or “pilot stud*” or “quasiexperiment*” or “quasi experiment*” or “evaluat*” or “time series” or “time point*” or “repeated measur*”)  *Indexes=SCI-EXPANDED, SSCI, A&HCI, ESCI Timespan=All years* | TX (“control* stud*” or “clinical trial*” or “random* control* trial*” or “cluster random* trial*” or “case control stud*” or “pre test posttest control group design” or “pretest” or “posttest” or “static group comparison” or “pilot stud*” or “quasiexperiment*” or “quasi experiment*” or “evaluat*” or “time series” or “time point*” or “repeated measur*”) |
| 6 | 1 and 2 and 3 and 4 and 5 | 1 and 2 and 3 and 4 and 5 | 1 and 2 and 3 and 4 and 5 |  | (S1 AND S2 AND S3 AND S4 AND S5) |
| 7 | limit 6 to (english and article) | limit 6 to (journal article and english) | limit 6 to (english and journal article) | (#5 AND #4 AND #3 AND #2 AND #1) AND LANGUAGE: (English) AND DOCUMENT TYPES: (Article)  *Indexes=SCI-EXPANDED, SSCI, A&HCI, ESCI Timespan=All years* | S1 AND S2 AND S3 AND S4 AND S5    Limiters - Language: English |
